# Rapid kit-based ^68^Ga-labelling and PET imaging with THP-Tyr^3^-octreotate: a preliminary comparison with DOTA-Tyr^3^-octreotate

**DOI:** 10.1186/s13550-015-0131-1

**Published:** 2015-10-09

**Authors:** Michelle T. Ma, Carleen Cullinane, Kelly Waldeck, Peter Roselt, Rodney J. Hicks, Philip J. Blower

**Affiliations:** Division of Imaging Sciences and Biomedical Engineering, King’s College London, 4th Floor Lambeth Wing, St Thomas’ Hospital, London, SE1 7EH UK; Peter MacCallum Cancer Centre, East Melbourne, Victoria Australia; Sir Peter MacCallum Department of Oncology, University of Melbourne, Parkville, Victoria Australia

**Keywords:** Gallium-68, Bifunctional chelator, Somatostatin, Molecular imaging, Peptide receptor, Octreotate, Hydroxypyridinone

## Abstract

**Background:**

Ge/^68^Ga generators provide an inexpensive source of a PET isotope to hospitals without cyclotron facilities. The development of new ^68^Ga-based molecular imaging agents and subsequent clinical translation would be greatly facilitated by simplification of radiochemical syntheses. We report the properties of a *tris*(hydroxypyridinone) conjugate of the SSTR2-targeted peptide, Tyr^3^-octreotate (TATE), and compare the ^68^Ga-labelling and biodistribution of [^68^Ga(THP-TATE)] with the clinical radiopharmaceutical [^68^Ga(DOTATATE)].

**Methods:**

A *tris*(hydroxypyridinone) with a pendant isothiocyanate group was conjugated to the primary amine terminus of H_2_N-PEG_2_-Lys(iv-Dde)^5^-TATE, and the resulting conjugate was deprotected to provide THP-TATE. THP-TATE was radiolabelled with ^68^Ga^3+^ from a ^68^Ge/^68^Ga generator. *In vitro* uptake was assessed in SSTR2-positive 427-7 cells and SSTR2-negative 427 (parental) cells. Biodistribution of [^68^Ga(THP-TATE)] was compared with that of [^68^Ga(DOTATATE)] in Balb/c nude mice bearing SSTR2-positive AR42J tumours. PET scans were obtained 1 h post-injection, after which animals were euthanised and tissues/organs harvested and counted.

**Results:**

[^68^Ga(THP-TATE)] was radiolabelled and formulated rapidly in <2 min, in ≥95 % radiochemical yield at pH 5–6.5 and specific activities of 60–80 MBq nmol^−1^ at ambient temperature. [^68^Ga(THP-TATE)] was rapidly internalised into SSTR2-positive cells, but not SSTR2-negative cells, and receptor binding and internalisation were specific. Animals administered [^68^Ga(THP-TATE)] demonstrated comparable SSTR2-positive tumour activity (11.5 ± 0.6 %ID g^−1^) compared to animals administered [^68^Ga(DOTATATE)] (14.4 ± 0.8 %ID g^−1^). Co-administration of unconjugated Tyr^3^-octreotate effectively blocked tumour accumulation of [^68^Ga(THP-TATE)] (2.7 ± 0.6 %ID g^−1^). Blood clearance of [^68^Ga(THP-TATE)] was rapid and excretion was predominantly renal, although compared to [^68^Ga(DOTATATE)], [^68^Ga(THP-TATE)] exhibited comparatively longer kidney retention.

**Conclusions:**

Radiochemical synthesis of [^68^Ga(THP-TATE)] is significantly faster, proceeds under milder conditions, and requires less manipulation than that of [^68^Ga(DOTATATE)]. A ^68^Ga-labelled *tris*(hydroxypyridinone) conjugate of Tyr^3^-octreotate demonstrates specificity and targeting affinity for SSTR2 receptors, with comparable *in vivo* targeting affinity to the clinical PET tracer, [^68^Ga(DOTATATE)]. Thus, peptide conjugates based on *tris*(hydroxypyridinones) are conducive to translation to kit-based preparation of PET tracers, enabling the expansion and adoption of ^68^Ga PET in hospitals and imaging centres without the need for costly automated synthesis modules.

## Background

The positron-emitting isotope gallium-68 (^68^Ga) possesses decay properties suitable for PET imaging (*t*_½_ = 68 min, 90 % positron yield, 1.9 MeV) and has been utilised in peptide receptor-targeted radiopharmaceuticals including somatostatin- [1–5], PSMA- [6], GRPR- [7] and GLP-1R-targeted [8] conjugates. Such agents are important as diagnostic agents in theranostic pairs of pharmaceuticals in personalised medicine [9]. The ^68^Ge/^68^Ga generator (^68^Ge *t*_½_ = 270 days) provides hospitals daily access to ^68^Ga without expensive cyclotron facilities, and a European pharmaceutical-grade generator has recently received marketing authorisation [10]. In terms of simplicity and accessibility, the ^68^Ge/^68^Ga generator has the potential to become the “PET equivalent” of the ^99^Mo/^99m^Tc generator, provided that suitable kit-based chemistry can be developed to facilitate clinical translation.

The macrocyclic chelator DOTA is frequently employed as a chelator for stable coordination of ^68^Ga^3+^, but synthesis of this complex requires heating at 80–100 °C for 5–10 min at pH 3–5 (although microwave irradiation can reduce reaction times to 1 min [11]), and often requires a post-synthetic purification step [11–18]. As such, it is not optimal for rapid kit-based syntheses of ^68^Ga-labelled radiotracers. Ideally, kit-based synthesis of such tracers could make use of a chelator that coordinates ^68^Ga^3+^ rapidly (<2 min) at ambient temperature to minimise synthesis time and simplify labelling and formulation procedures. Alternative chelators for ^68^Ga^3+^ have been reported, including NOTA/NODAGA [19–22], TRAP and its derivatives [23–26], sarcophagines [27], HBED and its derivatives [6, 28], substituted 6-amino-perhydrodiazepines AAZTA [29], the siderophore FSC [30], and a series of chelators based on substituted pyridine carboxylates (DEDPA) [31–33]. The bifunctional chelator HBED-CC is used clinically in the peptide-based ^68^Ga-labelled radiopharmaceutical, ^68^Ga-HBED-PSMA, which targets the prostate specific membrane antigen expressed in metastatic prostate cancer [6]. Derivatives of HBED, along with TRAP, NOTA, AAZTA, FSC and DEDPA conjugates have demonstrated desirable radiolabelling properties, with labelling proceeding rapidly at room temperature in all cases. We have reported that a tripodal *tris*(hydroxypyridinone) ligand coordinates ^68^Ga^3+^ via six *O*-atoms at mild pH (pH 6.5–7.0), at low ligand concentrations (10 μM) in <5 min, and specific activities of up to 80 MBq nmol^−1^ [34]. Bifunctional ^68^Ga-labelled derivatives of this compound are stable to demetallation *in vivo*, accumulate selectively in target tissue and are excreted mainly via a renal route [35].

**Chart 1:**
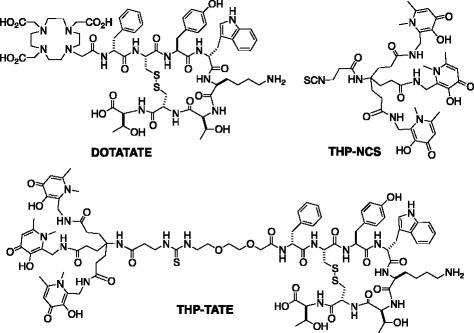
Structures of DOTATATE, the bifunctional chelator, THP-NCS and the new peptide conjugate, THP-TATE

We now report the synthesis, simple ^68^Ga-labelling and biodistribution of a somatostatin-2 receptor (SSTR2)-targeting *tris*(hydroxypyridinone) conjugate, THP-TATE. The SSTR2-targeting radiopharmaceutical [^68^Ga(DOTATATE)] has demonstrated superior clinical resolution and sensitivity compared to the ^111^In-labelled SPECT tracer, [^111^In(DTPA-octreotide)], in identifying tumours expressing SSTR2 in neuroendocrine cancer patients [3]. Despite the multistep radiochemistry required, [^68^Ga(DOTATATE)] is used routinely in PET clinics, and in conjunction with ^18^F-FDG [5], is important in determining therapeutic regimes of patients presenting with neuroendocrine tumours [1, 2, 4, 9, 10]. It is instructive to compare DOTATATE with THP-TATE (Chart 1), both in terms of (i) radiosynthesis in a hospital radiopharmacy and (ii) preclinical biodistribution, in order to evaluate the advantages and disadvantages of this new class of *tris*(hydroxypyridinone) chelators.

## Methods

### Materials and instrumentation

Mass spectra were recorded on an Agilent 6510 Q-TOF LC/MS mass spectrometer (Agilent, Palo Alto, CA). Instant thin-layer chromatography strips (ITLC-SG) were obtained from Varian Medical Systems UK, Ltd. (Crawley, UK), and ITLC strips were visualised using a Raytest Rita-Star TLC scanner. Semi-preparative reverse-phase HPLC was conducted using an Agilent Eclipse XDB-C18 column (9.4 × 250 mm, 5 μm) coupled to an Agilent 1200 LC system, with a 3 mL min^−1^ flow rate and UV spectroscopic detection at 220 nm. Mobile phase A contained water with 0.2 % TFA, and mobile phase B contained acetonitrile with 0.2 % TFA. The gradient started with 100 % A at 0 min, and the concentration of B increased at a rate of 1 % min^−1^.

Analytical reverse-phase HPLC and radio-HPLC traces were acquired using two different instruments: (1) an Agilent 1200 LC system with an Agilent Zorbax Eclipse XDB-C18 column (4.6 × 150 mm, 5 μm) and UV spectroscopic detection at 220 nm. The radio-HPLC was coupled to a LabLogic Flow-Count detector with a sodium iodide probe (B-FC-3200). Mobile phase A comprised water with 0.1 % TFA, and mobile phase B comprised acetonitrile with 0.1 % TFA. For method 1, the concentration of B increased at a rate of 1.67 % min^−1^, with 100 % A at 0 min and 50 % B at 30 min with a flow rate of 1 mL min^−1^; (2) an Agilent Zorbax Eclipse XDB-C18 column (4.6 × 150 mm, 5 μm) with a 1 mL min^−1^ flow rate and UV spectroscopic detection at 220 nm coupled to a Shimadzu HPLC. This was coupled to a radiation detector consisting of an Ortec model 276 Photomultiplier Base with Preamplifier, Amplifier, BIAS supply and SCA and a Bicron 1M 11.2 Photomultiplier Tube. For method 2, the concentration of B increased at a rate of 6.67 % min^−1^, with 100 % A at 0 min and 80 % B at 12 min.

Analytical size-exclusion radio-HPLC traces were acquired using an Agilent 1200 Series HPLC system and a Phenomenex Biosep 2000 (300 × 7.8 mm) size-exclusion column with a phosphate-buffered saline mobile phase.

For initial radiolabelling and characterisation studies that utilised <400 MBq, an Eckert and Ziegler ^68^Ge/^68^Ga generator (Berlin, Germany) was used. For biodistribution studies, and experiments that utilised >600 MBq ^68^Ga, an iThemba Labs 1.85 GBq ^68^Ge/^68^Ga generator (IDB Holland BV, Netherlands) was used.

### Synthesis of THP-TATE

The peptide H-PEG_2_-dPhe-Cys-Tyr-dTrp-Lys(iv-Dde)-Thr-Cys-Thr-OH was synthesised using standard solid-phase peptide synthesis protocols [36–39], cyclised using 2,2′-dithiodipyridine and purified using reverse-phase semi-preparative HPLC. PEG_2_-Lys(iv-Dde)^5^-TATE (5–6 mg) was dissolved in dimethylsulfoxide (100–300 μL) and added to THP-NCS (synthesised as previously described [35]) (4 mg) in dimethylsulfoxide (100–300 μL), and diisopropylethylamine (5–10 μL) was added. The reaction solution was heated in a microwave synthesiser (120 °C, 300 W, 30 min) and then applied to a reverse-phase HPLC column (conditions above). Fractions containing the desired (iv-Dde)-protected conjugate eluted at 45–47 min and were combined and lyophilised. MS: *m*/*z* [C_106_H_143_N_19_O_27_S_3_ + 3H]^3+^, observed monoisotopic peak = 737.66, calculated = 737.66; [C_106_H_143_N_19_O_27_S_3_ + 2H]^2+^, observed monoisotopic peak = 1105.99, calculated = 1105.99. The (iv-Dde)-protected conjugate was dissolved in a solution of 2 % hydrazine in dimethylformamide (1–2 mL). Within 30 min, the solution was applied to a reverse-phase HPLC column, and fractions containing THP-TATE eluted at 33–35 min and were combined and lyophilised. MS: *m*/*z* [C_93_H_125_N_19_O_25_S_3_ + 3H]^3+^, observed monoisotopic peak = 668.95, calculated = 668.95; [C_93_H_125_N_19_O_25_S_3_ + 2H]^2+^, observed monoisotopic peak = 1002.92, calculated = 1002.92, isolated yield ~25 %. Analytical HPLC (220 nm): RT (retention time) = 18.97 min, >97 % purity (method 1, see above).

### Radiolabelling

Initial radiolabelling experiments utilised an Eckert and Ziegler ^68^Ge/^68^Ga generator. Aqueous HCl solution (0.1 M, 5 mL) was passed through the generator, and the eluate was fractionated (5 × 1 mL). The second fraction (1 mL, containing 90–100 MBq ^68^Ga) was added directly to an ethanol/water solution (50 %/50 %, 50 μL) of THP-TATE (25 μg) and immediately followed by a solution of ammonium acetate (2 M, 200 μL) to obtain a solution of pH 5–6. This solution was immediately applied to an analytical reverse-phase C18 HPLC column. [^68^Ga(THP-TATE)]: radiochemical yield >99 % (HPLC), HPLC: RT = 20.23 min (HPLC method 1).

The non-radioactive analogue, [^*nat*^Ga(THP-TATE)] was also prepared. An aqueous solution of Ga(NO_3_)_3_ (1 mg mL^−1^, 4 mM, 5 μL) was added to THP-TATE (25 μg) dissolved in deionised water/ethanol (50 %/50 %, 50 μL). The solutions were applied to an analytical reverse-phase C18 HPLC column as well as being subjected to LCMS analysis. [^*nat*^Ga(THP-TATE)]: HPLC RT = 20.17 min (HPLC method 1); [C_93_H_122_N_19_O_25_S_3_Ga + 3H]^3+^, observed monoisotopic peak = 690.92, calculated = 690.92; [C_93_H_122_N_19_O_25_S_3_Ga + 2H]^2+^, observed monoisotopic peak = 1035.87, calculated = 1035.87.

For *in vivo* and *in vitro* studies, generator-produced ^68^Ga^3+^ (800–1000 MBq, iThemba Labs generator) was concentrated on an AG 50WX4 (400 mesh) cation exchange cartridge and eluted with 200 μL 0.9 M HCl in ethanol/water (90 %/10 %) [16]. This volume was diluted in deionised water (800 μL) and directly added to THP-TATE (25 μg) at ambient temperature, followed immediately by addition of aqueous ammonium acetate (2 M, 400 μL) to obtain solutions of pH ~6.5, resulting in [^68^Ga(THP-TATE)]. These solutions were further diluted by addition of saline solution (0.9 % NaCl *w*/*v*, 1.1 mL). Within 2–5 min of addition of ^68^Ga^3+^ to the conjugates, the solutions were subjected to analytical reverse-phase HPLC and ITLC analysis. [^68^Ga(THP-TATE)]: radiochemical yield >95 % (ITLC), HPLC: RT = 10.48 min (HPLC method 2).

Synthesis of [^68^Ga(DOTATATE)] was undertaken using methods previously reported [17]. Briefly, an iThemba Labs generator at approximately 3 months post-calibration was eluted with aqueous HCl (0.4 M, 5 mL). The eluate was passed through an AG 50WX8 (400 mesh) cation exchange resin, and the ^68^Ga^3+^ was retained on the resin. The resin was washed with a solution of 80 % acetone/0.15 N HCl (1 mL) to remove residual ^68^Ge breakthrough, followed by elution of ^68^Ga^3+^ (using a solution of 97.6 % acetone/0.05 N HCl, 400 μL) into a pre-heated reaction vial containing DOTATATE (42 μg), ascorbic acid and gentisic acid in sterile Milli-Q water (5 mL). After 10 min at 105 °C, the reaction mixture was passed through a reverse-phase solid-phase extraction cartridge (Strata-X, 30 mg, Phenomenex). The Strata-X cartridge was rinsed with sterile Milli-Q water, and [^68^Ga(DOTATATE)] was subsequently recovered with ethanol (500 μL). The ethanol solution containing [^68^Ga(DOTATATE)] was transferred into a vial containing saline for injection (9 mL), and the resultant mixture passed through a low protein-binding filter. Radiochemical yields ranged from 50 to 70 %, and radiochemical purity was greater than 95 %.

### Log *P*_OCT/PBS_ determination

A solution containing [^68^Ga(THP-TATE)] (10 μL, synthesised using eluate from an Eckert and Ziegler generator as described above) was added to 500 μL of octanol and 490 μL of aqueous phosphate-buffered saline solution. The mixture was agitated using a vortex for 3–4 min, and the phases separated by centrifugation (4000 rpm, 5 min). Aliquots from each phase (50 μL) were counted for radioactivity in a gamma counter. The experiment was repeated six times.

### Serum stability

A solution containing [^68^Ga(THP-TATE)] (150 μL, synthesised using eluate from an Eckert and Ziegler generator as described above) was added to 1.5 mL of fresh human female O^+^ serum, incubated at 37 °C for 5 h, and the reaction mixture was analysed using size-exclusion HPLC chromatography. Concurrently, a solution of ^68^Ga^3+^ in ammonium acetate (0.33 M, 8 MBq, 300 μL) was added to 1.5 mL of serum and incubated at 37 °C for 4 h, followed by analysis using size-exclusion HPLC.

### *In vitro* uptake

The A427 human non-small cell lung carcinoma cell line was obtained from American Type Culture Collection (catalogue number: HTB-53). The SSTR2 over-expressing cell line A427-7 was a gift from Prof. Buck Rogers [40]. A427-7 and parental A427 cells were plated in Minimum Essential Medium (MEM) containing 10 % FBS at 5 × 10^5^ cells per well in poly-d-lysine-coated 12-well cell culture dishes for 24 h. On the day of the binding assay, cells were washed in PBS and equilibrated in MEM containing 1 % FCS. Cells were then treated with [^68^Ga(THP-TATE)] (1.5 MBq, 5 μL, 4 μM THP-TATE), with or without blocking TATE peptide (5 μL, 800 μM, 200-fold excess) for 5, 15, 30 and 60 min (A427-7 cells) and 60 min (A427 parental cells) in triplicate. Uptake was terminated by placing the cells on ice. Unbound free tracer was collected, with the supernatant and cold PBS washes combined for this fraction. The surface-bound tracer fraction was collected through two 10-min acid washes (0.1 M glycine in saline, pH 2.3). Finally, the internalised fraction was collected through incubation in 1 M NaOH for 10 min. The activity of these fractions was determined using a gamma counter (Biomedex). Protein concentration in each well was determined using the Pierce BCA Protein Assay Kit (Amersham) on the internalised fractions collected. Results were calculated as a percentage of added radioactivity and normalised to protein concentration. The experiment was repeated three times.

### PET scanning and biodistribution

All animal experiments were performed with approval from the Peter MacCallum animal ethics committee. Six- to eight-week-old Balb/c nude mice (Animal Resources Centre, Western Australia) were implanted subcutaneously on the right flank with three million AR42J cells (sourced from ATCC). Once the tumours reached a volume >150 mm^3^, the animals (*n* = 3) were injected intravenously with 23–28 MBq [^68^Ga(THP-TATE)] (containing 1 μg of THP-TATE). For blocking studies, animals (*n* = 3) were coinjected with Tyr^3^-octreotate peptide (400 μg). For [^68^Ga(DOTATATE)], the animals (*n* = 3) were injected with 8 MBq of the tracer (containing 1 μg of DOTATATE). At 1 h, the animals were anaesthetised and imaged on a Philips MOSAIC small animal PET scanner. The images were reconstructed using a 3D RAMLA algorithm and tracer uptake determined as described previously [41]. On completion of the scan, animals were euthanised and tissues harvested, weighed and radioactivity counted using a gamma counter (Biomedex). Quantitation of PET images was performed using in-house software (MARVn 3.31). Regions of interest were drawn around tissues of interest and uptake ratio calculated as the maximum pixel intensity in the tumour divided by the average uptake in a mediastinal background region, liver or kidneys, as appropriate.

## Results

### Synthesis and radiolabelling of THP-TATE

Reaction of the bifunctional chelator THP-NCS (Chart 1) with H_2_N-PEG_2_-Lys(iv-Dde)^5^-TATE under microwave conditions resulted in the facile formation of THP-PEG_2_-Lys(iv-Dde)^5^-TATE. Removal of the iv-Dde group from the Lys^5^ side-chain resulted in the formation of THP-TATE (Chart 1).

The new THP-TATE peptide conjugate could be radiolabelled with generator-produced eluate that was added directly from the generator or eluate that was preconditioned to concentrate activity and remove any contaminating ^68^Ge [16]. In both cases, ^68^Ga^3+^ in 1 mL HCl solution was added to THP-TATE (10 nmol) at ambient temperature, followed immediately by addition of aqueous ammonium acetate and saline to obtain solutions of pH 5–7, which were then immediately subjected to ITLC and HPLC analysis. This synthetic protocol reproducibly provided the labelled conjugate [^68^Ga(THP-TATE)] in >95 % radiochemical yield (with <5 % attributable to unchelated ^68^Ga^3+^) and in the case where a generator eluting 750–1000 MBq was utilised, specific activities of 60–80 MBq nmol^−1^. Using lower quantities of THP-TATE (5 nmol) resulted in radiochemical yields of 80–90 %, indicating that for every 1 mL solution containing ^68^Ga^3+^, at least 25 μg of THP-TATE is required to reliably achieve radiochemical yields >95 %. Without addition of ammonium acetate solution, radiolabelling of THP-TATE was not observed: complex formation did not occur in highly acidic solutions (such as in the final solution isolated after preconditioning the eluate (0.9 M HCl) or that used to elute the generator (0.1 M HCl)).

**Fig. 1:**
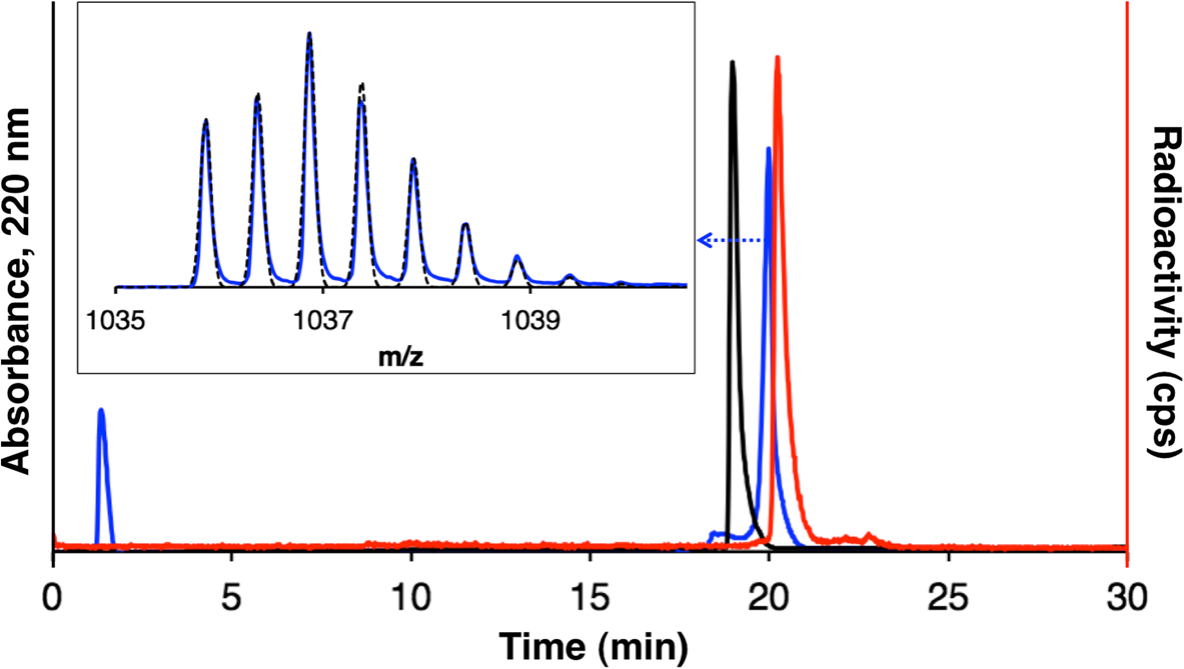
HPLC traces (*λ*_220_) of THP-TATE (*black*) and [^*nat*^Ga(THP-TATE)] (*blue*) and radio-HPLC trace of [^68^Ga(THP-TATE)] (*red*). *Inset*: experimental (*blue*) and simulated (*black dashed*) mass spectral signal of [^*nat*^Ga(THP-TATE) +2H]^2+^

HPLC and LCMS analyses of the analogous non-radioactive [^*nat*^Ga(THP-TATE)] compound were undertaken to verify the identity of the radiolabelled product. Only a single product was observed in the total ion chromatogram of the LCMS of [^*nat*^Ga(THP-TATE)]. Only two signals were observed in the resulting mass spectrum, corresponding to the dipositive and tripositive ions of [^*nat*^Ga(THP-TATE)] (Fig. 1, inset). Under the HPLC conditions employed, [^68^Ga(THP-TATE)] possessed a retention time (RT) of 20.23 min (sodium iodide scintillation detection) (Fig. 1, red trace). Non-radioactive [^*nat*^Ga(THP-TATE)] possessed a RT of 20.17 min (UV detection at 220 nm) (Fig. 1, blue trace), with the difference in retention times a result of the configuration of the detectors in series. The co-elution of the non-radioactive and radioactive Ga^3+^-labelled peptides was indicative of the formation of a single radiolabelled product (>95 % radiochemical purity) where the Ga^3+^:THP-TATE stoichiometry = 1:1.

### Lipophilicity and serum stability studies

The log *P*_OCT/PBS_ of [^68^Ga(THP-TATE) measured −3.20 ± 0.09 (*n* = 6), almost 0.5 units higher than that of [^68^Ga(DOTATATE)] which possesses a log *P*_OCT/PBS_ of −3.69 [42], indicating that the Ga^3+^-coordinated THP complex is significantly more lipophilic than the DOTA complex.

**Fig. 2:**
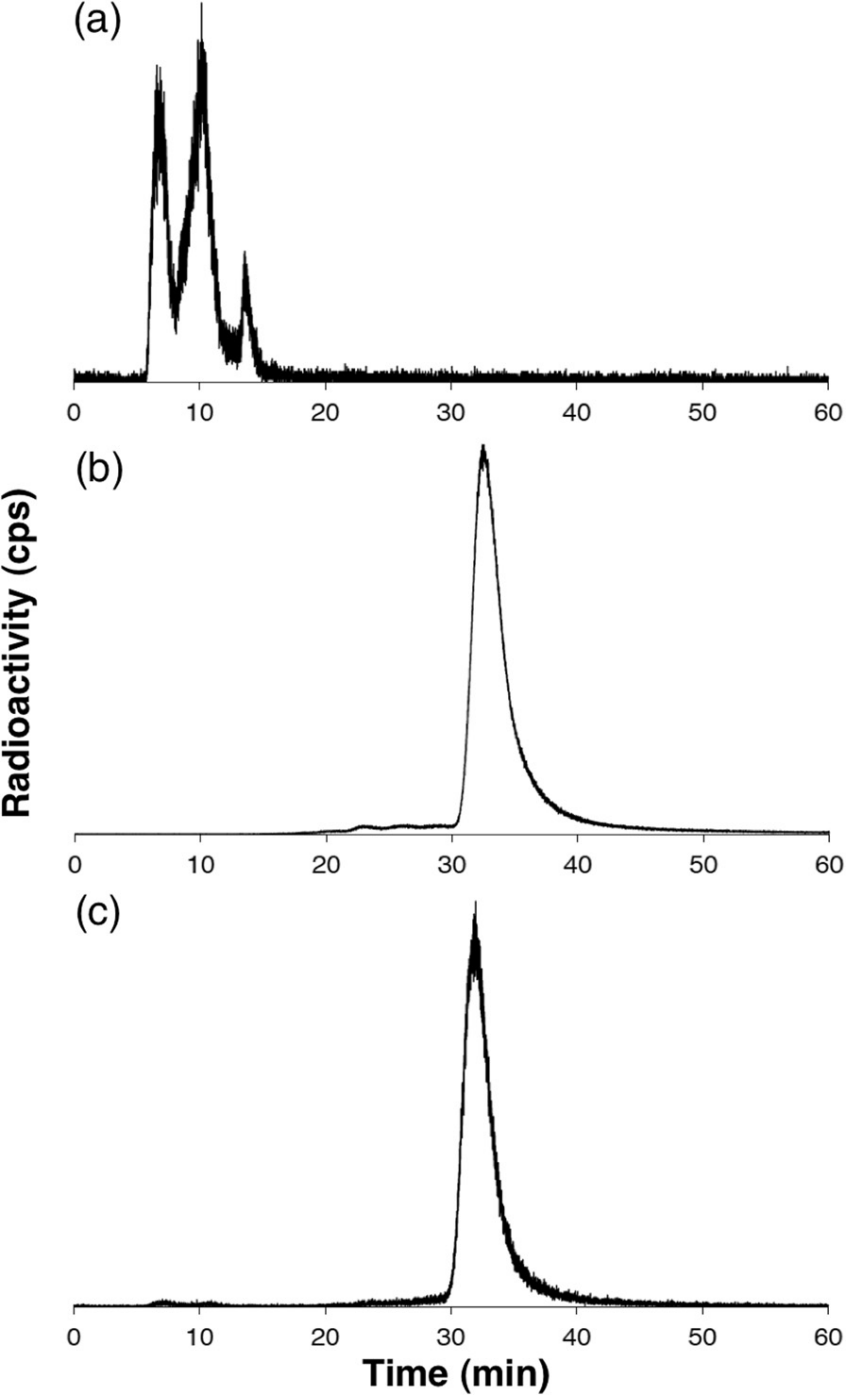
Size-exclusion HPLC chromatograms of **a** a sample of [^68^Ga(acetate)_3_] incubated in human serum for 4 h, **b** [^68^Ga(THP-TATE)] and **c** [^68^Ga(THP-TATE)] after 5 h incubation in human serum at 37 °C

Serum stability studies were undertaken to determine whether [^68^Ga(THP-TATE)] releases ^68^Ga^3+^ to endogenous serum proteins. Addition of generator-produced ^68^Ga^3+^ to a solution of human serum resulted in ^68^Ga-bound protein adducts that possessed distinct retention times of 6.6, 10.4 and 13.8 min when applied to the size-exclusion HPLC column utilised in this study (Fig. 2a). Radiolabelled [^68^Ga(THP-TATE)] possessed a retention time of 31.8 min (Fig. 2b). After incubation of [^68^Ga(THP-TATE)] in fresh human serum at 37 °C for 5 h, the size-exclusion chromatogram exhibited a strong signal at the same retention time of [^68^Ga(THP-TATE)] (>98 % integration), as well as small signals between 5 and 15 min (<2 % integration), indicating that less than 2 % of ^68^Ga^3+^ bound to THP-TATE underwent transchelation to serum proteins (Fig. 2c) during 5 h.

### *In vitro* cell binding and internalisation of [^68^Ga(THP-TATE)]

**Fig. 3:**
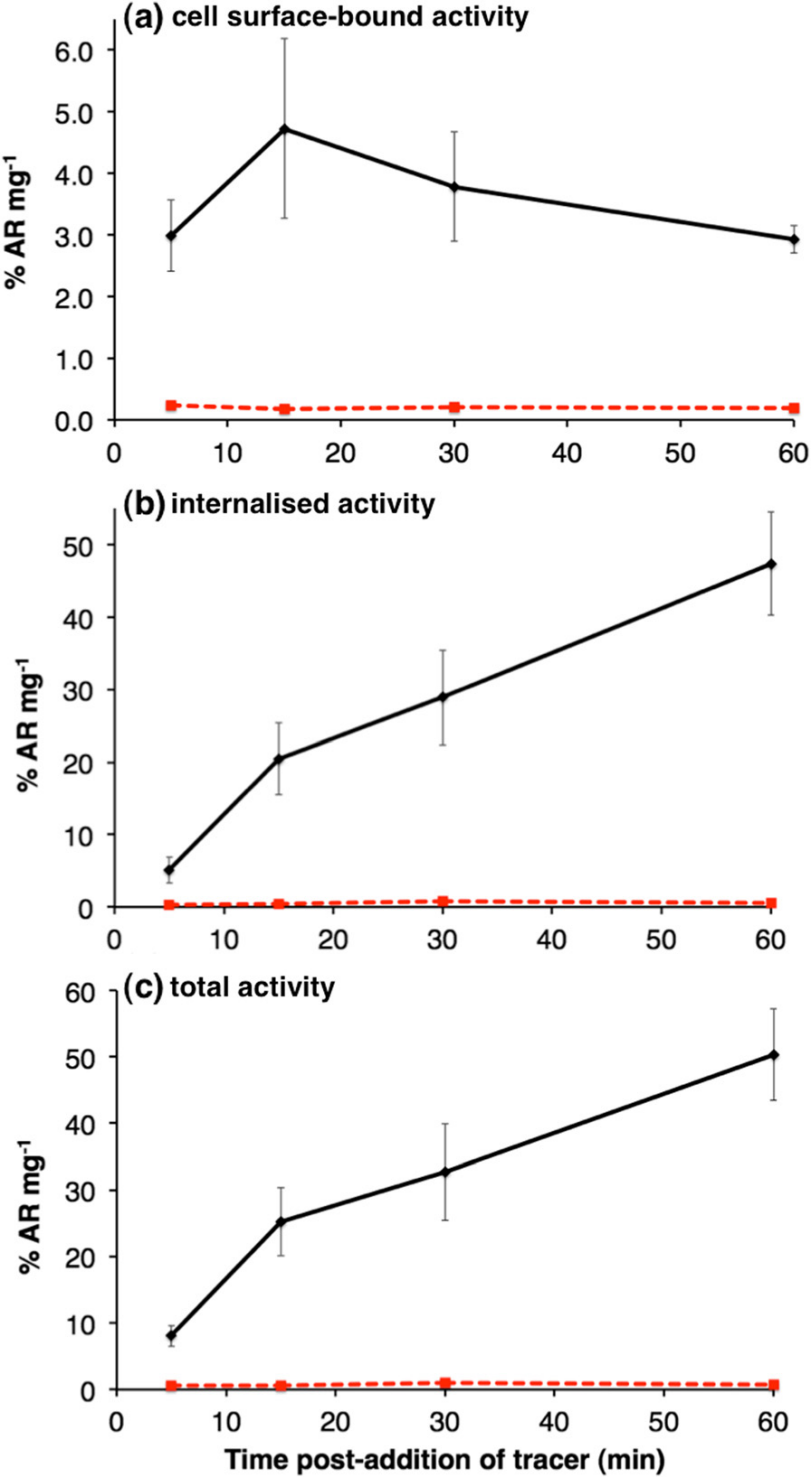
SSTR2-positive A427-7 cell uptake of [^68^Ga(THP-TATE)] (*black*) and [^68^Ga(THP-TATE)] in the presence of excess TATE peptide (*red*). **a** Cell surface-bound activity, **b** internalised activity and **c** total activity associated with cells. Uptake is expressed as a percentage of added radioactivity (AR)/mg of protein, with uptake representing the mean from three separate experiments. *Error bars* correspond to standard error of the mean

To assess the internalisation of [^68^Ga(THP-TATE)], and specificity of [^68^Ga(THP-TATE)] for SSTR2 receptors, [^68^Ga(THP-TATE)] was incubated with SSTR2-positive A427-7 cells [41]. At 5, 15, 30 and 60 min after addition of [^68^Ga(THP-TATE)], the amount of surface-bound and internalised radioactivity was quantified (Fig. 3). After a 60-min incubation, <4 % of added radioactivity/mg of protein (%AR mg^−1^) was bound to the cell surface, but over 40 %AR mg^−1^ was internalised. Indeed, at all time points, surface-bound activity measured <10 %AR mg^−1^ whilst internalised activity increased over the course of the 60-min experiment.

A427-7 cells were also co-incubated with [^68^Ga(THP-TATE)] and an excess of unconjugated Tyr^3^-octreotate (TATE, 200-fold excess compared to THP-TATE) peptide to determine SSTR2-specific uptake [41]. At all time points, internalised and surface-bound activity measured <1 %AR mg^−1^ (Fig. 3). Lastly, [^68^Ga(THP-TATE)] was incubated with the SSTR2-negative A427 parental cell line. After a 60-min incubation with [^68^Ga(THP-TATE)], uptake (surface-bound and internalised) in A427 cells measured 0.15 ± 0.04 %AR mg^−1^ vs uptake in A427-7 cells, which measured 50.4 ± 6.9 %AR mg^−1^.

### Biodistribution of [^68^Ga(THP-TATE)] and [^68^Ga(DOTATATE)]

The biodistribution of [^68^Ga(THP-TATE)] was assessed in Balb/c nu/nu mice bearing SSTR2-positive AR42J tumours. Each animal was administered [^68^Ga(THP-TATE)] and PET scanned at 1 h post-injection (PI) for 10 min, followed by euthanasia and organ harvesting for *ex vivo* radioactivity counting. To assess specificity of the radiotracer, a separate group of animals was co-administered [^68^Ga(THP-TATE)] and TATE peptide, followed by scanning, euthanasia and *ex vivo* organ counting 1 h PI. To allow for comparison between the biodistribution of [^68^Ga(THP-TATE)] and [^68^Ga(DOTATATE)], a third group of animals was administered [^68^Ga(DOTATATE)] followed by scanning, euthanasia and *ex vivo* organ counting 1 h PI.

**Fig. 4:**
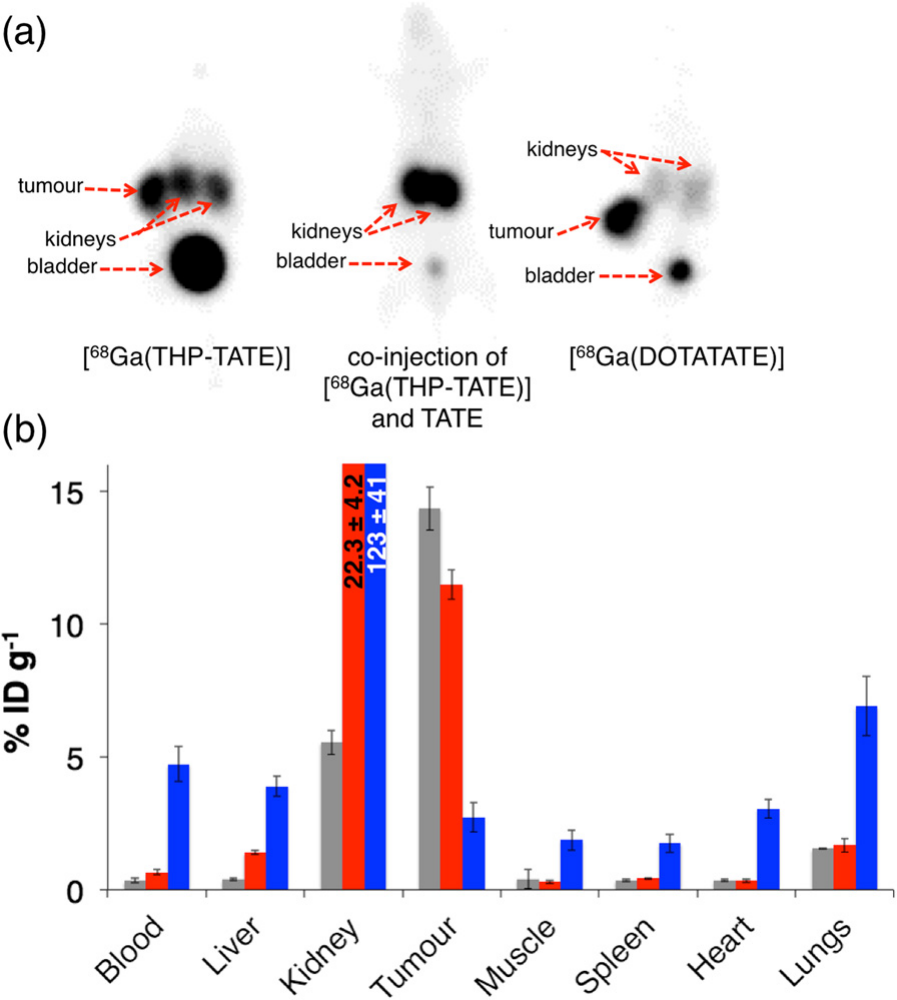
**a** Whole-body PET maximum intensity projection of Balb/c nu/nu mice bearing an AR47J tumour on the right flank 1 h PI of ^68^Ga-labelled tracers. **b** Biodistribution of mice administered [^68^Ga(DOTATATE)] (*grey*) and [^68^Ga(THP-TATE)] (*red*) and co-administered [^68^Ga(THP-TATE)] and TATE (blocked, *blue*) 1 h PI; *n* = 3 and *error bars* correspond to standard error of the mean

In PET scans of animals administered [^68^Ga(THP-TATE)] (Fig. 4a), the tumour of each animal could be clearly delineated, as well as the kidneys. The tumour to background (mediastinum), liver and kidney ratios are listed in Table 1. Excretion was largely renal, with significant amounts of activity in the bladders of all animals at 1 h PI. In contrast, tumours in animals co-administered TATE peptide could not be delineated. Animals administered [^68^Ga(DOTATATE)] exhibited higher tumour to kidney, tumour to liver, and tumour to background ratios than those of [^68^Ga(THP-TATE)] (Table 1).

**Table 1:** Tumour to organ/background ratios (±SEM) obtained from PET images of animals administered [^68^Ga(DOTATATE)] and [^68^Ga(THP-TATE)] (*n* = 3)

	[^68^Ga(DOTATATE)]	[^68^Ga(THP-TATE)]
Tumour to kidney	5.7 ± 0.2	1.5 ± 0.5
Tumour to liver	27.2 ± 3.9	10.5 ± 2.0
Tumour to mediastinum	51.2 ± 3.8.	36.0 ± 8.1

*Ex vivo* biodistribution data were consistent with PET data (Fig. 4b). AR42J tumour uptake in animals administered [^68^Ga(THP-TATE)] (11.5 ± 0.6 %ID g^−1^) was slightly lower than tumour uptake in animals administered [^68^Ga(DOTATATE)] (14.4 ± 0.8 %ID g^−1^, mean difference = 2.9 %ID g^−1^, 95 % confidence interval (CI) = 0.6–5.1 %ID g^−1^, *p* = 0.023). Kidney retention in the [^68^Ga(THP-TATE)] group was significantly higher (22.3 ± 4.2 %ID g^−1^) compared to that in the [^68^Ga(DOTATATE)] group (5.6 ± 0.5 %ID g^−1^, mean difference = 16.7 %ID g^−1^, 95 % CI = 7.1–26.3 %ID g^−1^, *p* = 0.0085). Additionally, increased liver accumulation was observed for [^68^Ga(THP-TATE)] compared to [^68^Ga(DOTATATE)] (1.4 ± 0.1 vs 0.4 ± 0.04 %ID g^−1^, respectively, mean difference = 1.0 %ID g^−1^, 95 % CI = 0.8–1.2 %ID g^−1^, *p* = 0.00010) as well as higher blood retention (0.6 ± 0.1 vs 0.3 ± 0.08 %ID g^−1^, respectively, mean difference = 0.3 %ID g^−1^, 95 % CI = 0.004–0.603 %ID g^−1^, *p* = 0.048) 1 h PI.

Compared to animals administered solely [^68^Ga(THP-TATE)], animals co-administered [^68^Ga(THP-TATE)] and TATE peptide demonstrated lower uptake in tumours (11.5 ± 0.6 vs 2.7 ± 0.6 %ID g^−1^, respectively, mean difference = 8.8 %ID g^−1^, 95 % CI = 7.0–10.5 %ID g^−1^, *p* = 0.00016), very high kidney retention (22.3 ± 4.2 vs 123.3 ± 41.2 %ID g^−1^, mean difference = 101.1, 95 % CI = 7.1–195.0 %ID g^−1^, *p* = 0.040) and higher blood activity (0.6 ± 0.1 vs 4.7 ± 0.7 %ID g^−1^, mean difference = 4.1 %ID g^−1^, 95 % CI = 2.6–5.6 %ID g^−1^, *p* = 0.0017), and significantly higher activity values were associated with non-target organs and tissue (Fig. 4b).

## Discussion

The work described here demonstrates that with suitable design of chelators—in this case, the tripodal hexadentate THP chelator—to facilitate extremely fast chelation under mild conditions and low ligand concentration, rapid kit-based synthesis of ^68^Ga radiopharmaceuticals is readily achievable and can be performed in a few minutes using a generator, a kit vial, a syringe and appropriate shielding. This has the potential to greatly increase the availability of ^68^Ga radiopharmaceuticals for the benefit of more hospitals and patients.

Several methods for radiosynthesis of [^68^Ga(DOTATATE)] have been reported, and although radiochemical yields of between >99 and 95 % can be obtained (obviating a post-synthetic purification step), all require 5–10-min reaction time at 80–100 °C [11–18] or microwave heating for 1 min at 90 °C [11] with pH 3–5 (Table 2). Radiochemical syntheses typically require between 7 and 30 nmol of DOTATATE (or DOTATOC), although in the case of microwave heating, 0.5–1 nmol of conjugate is sufficient for quantitative radiolabelling [11–18].

**Table 2:** Comparison of ^68^Ga^3+^ labelling conditions commonly employed for radiosynthesis of [^68^Ga(DOTATATE)] and the conditions employed for radiosynthesis of [^68^Ga(THP-TATE)]

Reaction variables	[^68^Ga(DOTATATE)]	[^68^Ga(THP-TATE)]
Temperature	80–90 °C	20–25 °C
Time	5–10 min	<2 min
Yield	>95 %	>95 %
pH	3–5	5–7
Amount of conjugate	7–30 nmol	10 nmol

In contrast, radiosynthesis to produce [^68^Ga(THP-TATE)] in specific activities sufficient for *in vivo* administration could be undertaken in <2 min, at room temperature and formulated to pH 6–7 at the same time the reaction occurs (Table 2). Under the conditions employed here, it is possible that the rate of reaction of ^68^Ga^3+^ with THP-TATE is limited only by the rate of diffusion of components in the reaction mixture. Provided 25 μg (equivalent to 10 nmol) of THP-TATE is utilised, radiochemical yields >95 % are routinely achievable. The specific activities achieved (60–80 MBq nmol^−1^) are comparable to specific activities achieved in the clinical production of [^68^Ga(DOTATATE)].

Several other chelators are capable of achieving near-quantitative radiochemical labelling at room temperature. NOTA/NODAGA conjugates can be radiolabelled at room temperature in radiochemical yields in excess of 95 % at pH 3.5–4 within 10 min [19]. The DEDPA chelator can similarly be radiolabelled in excess of 97 % yield at pH 4.5 in 10 min at nmol levels [31, 32]. Whilst TRAP and its derivatives have typically been labelled at elevated temperatures in order to achieve extraordinarily high specific activities, at pH 3.3, near quantitative-radiolabelling (~95 %) can be achieved at μM concentrations in 10 min at room temperature [24]. The advent of bifunctional *tris*(hydroxypyridinone) chelators increases the pH range at which biomolecules can be radiolabelled at room temperature, permitting labelling at neutral pH and hence ^68^Ga PET imaging of fusion proteins, antibody fragments and other proteins that are sensitive to extremes of heat and pH.

Whilst THP-TATE could be labelled using unprocessed, fractionated eluate directly from the generator, a post-processing method to remove any ^68^Ge (as required in radiopharmaceutical preparations from some ^68^Ge/^68^Ge generators) that ultimately provided an ethanolic solution (18 % ethanol in aqueous HCl solution) of ^68^Ga^3+^ was utilised in preparations of [^68^Ga(THP-TATE)] for *in vivo* experiments [16]. Radiochemical yields of [^68^Ga(THP-TATE)] prepared using such solutions were high, heating and post-purification were not required, and the final formulation was suitable for injection into mice (Table 2).

The bifunctional chelator THP-NCS provides a facile synthetic route to peptide conjugates bearing *tris*(hydroxypyridinone) chelators [35]. The THP chelator is significantly larger than DOTA, and previous work has suggested that increasing the distance between the THP chelator and the targeting peptide leads to increased receptor affinity [35]. A PEG linker was included in the THP-TATE conjugate to circumvent potential deleterious effects the close proximity of the THP group might exert upon the conjugate affinity for SSTR2 receptors. Synthesis from the Lys(iv-Dde)^5^ derivative, PEG_2_-Lys(Dde)^5^-TATE, ensured selective attachment of the isothiocyanate, THP-NCS, to the N-terminus of the peptide.

Less than 2 % of ^68^Ga^3+^ dissociated from [^68^Ga(THP-TATE)] to serum proteins in competition studies using fresh human serum over a 5-h incubation period, suggesting that [^68^Ga(THP-TATE)] is of sufficient stability to withstand competition from endogenous proteins *in vivo* over a time period of at least 1–2 h.

Similar to other agonist conjugates of Tyr^3^-octreotate [43–45], [^68^Ga(THP-TATE)] underwent rapid internalisation upon SSTR2 binding. Co-incubation of [^68^Ga(THP-TATE)] with an excess of TATE peptide effectively blocked binding of [^68^Ga(THP-TATE)] to SSTR2 receptors, and incubation of [^68^Ga(THP-TATE)] with SSTR-negative cells did not result in either surface-bound or internalised uptake of activity. These qualitative data strongly point to high specificity of [^68^Ga(THP-TATE)].

The biodistribution profile of [^68^Ga(THP-TATE)] demonstrated that, like [^68^Ga(DOTATATE)], [^68^Ga(THP-TATE)] targets SSTR2-positive tissue and is cleared predominantly via a renal pathway. Tumour uptake for [^68^Ga(THP-TATE)] and [^68^Ga(DOTATATE)] is comparable (11.5 ± 0.6 vs 14.4 ± 0.8 %ID g^−1^) but [^68^Ga(THP-TATE)] has a longer residence time in the kidney (22.3 ± 4.2 vs 5.6 ± 0.5 %ID g^−1^), higher uptake in the liver (1.4 ± 0.1 vs 0.4 ± 0.04 %ID g^−1^) and higher blood retention (0.6 ± 0.1 vs 0.3 ± 0.1 %ID g^−1^) 1 h PI, resulting in lower tumour to background/non-target organ ratios for [^68^Ga(THP-TATE)] compared to [^68^Ga(DOTATATE)] (Table 1). This is not a result of differences in charge, as the overall charge of both radiotracers is the same. Indeed, log *P*_OCT/PBS_ measurements indicated that [^68^Ga(THP-TATE)] is significantly more lipophilic than [^68^Ga(DOTATATE)] (by almost 0.5 units), and thus, it is most likely that differences in biodistribution, particularly liver uptake, arise from these differences in lipophilicity. We have observed similar *in vivo* behaviour for an α_v_β_3_ integrin-targeted conjugate of THP-NCS: [^68^Ga(THP-NCS-RGD)] activity measured 2.94 ± 0.06 %ID g^−1^ in the liver, 4.76 ± 0.36 %ID g^−1^ in the kidneys and 0.84 ± 0.09 %ID g^−1^ in the blood 1 h PI [35]. Lastly, although we did not detect colloids at the point of HPLC and ITLC analysis, the possibility that unchelated ^68^Ga^3+^ (<5 %), present in the formulation, resulted in some colloid formation between the point of analysis and the point of *in vivo* administration, in turn contributing to a small proportion of liver activity in animals administered [^68^Ga(THP-TATE)], cannot be completely eliminated.

PET scanning experiments and *ex vivo* biodistribution in animals co-administered TATE peptide (blockade group) demonstrated that TATE peptide effectively blocks SSTR2 receptor binding by [^68^Ga(THP-TATE)], indicating *in vivo* specificity of [^68^Ga(THP-TATE)] for SSTR2. Significantly higher blood and kidney activity in the blockade group was also observed, contrasting most [42, 45, 46] but not all [47] previous reports that compare preclinical biodistribution of SSTR2 radiotracers in blockade and non-blockade groups of SSTR2-positive tumour-bearing mice. It is possible that the significantly higher blood activity observed in the blockade group compared to the non-blockade group (4.7 ± 0.7 vs 0.6 ± 0.1 %ID g^−1^, respectively) is in part a consequence of persistent presence of the radiotracer in circulation in the absence of receptors available for binding, rather than high non-specific organ uptake. In this scenario, higher blood and kidney activity in the blocked group compared to that of the [^68^Ga(THP-TATE)] group is a result of blocked SSTR2 sites that are no longer able to function as a “sink” for [^68^Ga(THP-TATE)] [48]. It is also possible that the observed higher blood and kidney activity in the blockade group is a result of slower clearance of [^68^Ga(THP-TATE)] from circulation via a renal route in the presence of excess TATE peptide.

## Conclusions

Simplicity of labelling with minimal need for complex equipment and radiochemical expertise, which is likely to be a key to the wider availability of ^68^Ga PET, is afforded by appropriate design of the ^68^Ga chelator. The *tris*(hydroxypyridinone) bifunctional chelator, THP-NCS, provides facile access to the peptide conjugate THP-TATE, which can be radiolabelled with generator-produced ^68^Ga^3+^ in high radiochemical yield (>95 %) and specific activities of 60–80 MBq nmol^−1^. Radiosynthesis and formulation is rapid (<2 min), proceeds at ambient temperature and simply requires addition of ^68^Ga^3+^ solution to the conjugate and neutralisation with acetate solution. The resulting tracer, [^68^Ga(THP-TATE)], specifically binds to SSTR2 and, similar to other agonists of SSTR2, is rapidly internalised. *In vivo*, [^68^Ga(THP-TATE)] clears rapidly from circulation, accumulates specifically at SSTR2-positive tumours and is cleared predominantly via a renal pathway. In comparison with [^68^Ga(DOTATATE)], synthesis of [^68^Ga(THP-TATE)] is significantly faster and occurs at ambient temperature. [^68^Ga(THP-TATE)] and [^68^Ga(DOTATATE)] show comparable tumour uptake, but [^68^Ga(THP-TATE)] exhibits comparatively longer kidney retention.
